# Sex- and age-dependent susceptibility to ventricular arrhythmias in the rat heart ex vivo

**DOI:** 10.1038/s41598-024-53803-9

**Published:** 2024-02-11

**Authors:** Marta Oknińska, Monika Katarzyna Duda, Elżbieta Czarnowska, Joanna Bierła, Aleksandra Paterek, Michał Mączewski, Urszula Mackiewicz

**Affiliations:** 1 Department of Clinical Physiology, Centre of Postgraduate Medical Education, Marymoncka 99/103, 01-813 Warsaw, Poland; 2 Department of Pathology, The Children’s Memorial Health Institute, Aleja Dzieci Polskich 20, 04-736 Warsaw, Poland; 3 Department of Pathology, Medical University of Warsaw, Żwirki i Wigury 61, 02-091 Warsaw, Poland

**Keywords:** Ventricular arrhythmia, Sex differences, Aging, Ca^2+^ signaling, Fibrosis, Oxidative stress, Cardiology, Medical research

## Abstract

The incidence of life-threatening ventricular arrhythmias, the most common cause of sudden cardiac death (SCD), depends largely on the arrhythmic substrate that develops in the myocardium during the aging process. There is a large deficit of comparative studies on the development of this substrate in both sexes, with a particular paucity of studies in females. To identify the substrates of arrhythmia, fibrosis, cardiomyocyte hypertrophy, mitochondrial density, oxidative stress, antioxidant defense and intracellular Ca^2+^ signaling in isolated cardiomyocytes were measured in the hearts of 3- and 24-month-old female and male rats. Arrhythmia susceptibility was assessed in ex vivo perfused hearts after exposure to isoproterenol (ISO) and hydrogen peroxide (H_2_O_2_). The number of ventricular premature beats (PVBs), ventricular tachycardia (VT) and ventricular fibrillation (VF) episodes, as well as intrinsic heart rate, QRS and QT duration, were measured in ECG signals recorded from the surfaces of the beating hearts. After ISO administration, VT/VFs were formed only in the hearts of males, mainly older ones. In contrast, H_2_O_2_ led to VT/VF formation in the hearts of rats of both sexes but much more frequently in older males. We identified several components of the arrhythmia substrate that develop in the myocardium during the aging process, including high spontaneous ryanodine receptor activity in cardiomyocytes, fibrosis of varying severity in different layers of the myocardium (nonheterogenic fibrosis), and high levels of oxidative stress as measured by nitrated tyrosine levels. All of these elements appeared at a much greater intensity in male individuals during the aging process. On the other hand, in aging females, antioxidant defense at the level of H_2_O_2_ detoxification, measured as glutathione peroxidase expression, was weaker than that in males of the same age. We showed that sex has a significant effect on the development of an arrhythmic substrate during aging. This substrate determines the incidence of life-threatening ventricular arrhythmias in the presence of additional stimuli with proarrhythmic potential, such as catecholamine stimulation or oxidative stress, which are constant elements in the pathomechanism of most cardiovascular diseases.

## Introduction

Nearly half of people with cardiovascular disease die suddenly (SCD) from dangerous ventricular arrhythmias such as ventricular tachycardia (VT) or ventricular fibrillation (VF)^1^. Biological sex significantly affects the incidence and type of arrhythmia preceding SCD. The risk of SCD increases with age and is significantly greater in men. However, in women, SCD is often the first symptom of the disease, and taking any preventive action is more difficult^2,3^. The implantation of a cardioverter-defibrillator (ICD) is the main method of primary prevention of SCD. However, it has been shown that only 20–30% of patients with a device implanted for this purpose benefit from this therapy, while 70–80% of patients do not experience dangerous ventricular arrhythmias^4,5^. Moreover, more than 50% of the SCD victims did not meet the eligibility criteria for ICD implantation, which is a left ventricular ejection fraction < 30%^6^ and only 10% of all sudden death victims are deemed to be at high risk^1^. Therefore, there is an urgent need to develop more effective methods for predicting arrhythmia risk in patients of both sexes. This requires the identification of an arrhythmia substrate in the myocardium, the presence of which predisposes patients to life-threatening ventricular arrhythmias in the presence of stressors such as ischemia, reperfusion, or catecholamine stimulation.

VT and VF have been shown to arise most often by a re-entry mechanism^7^. The main triggers of re-entry are ventricular premature beats (PVBs), the formation of which is promoted by prolongation of the action potential (AP) and disturbances in intracellular calcium signaling due to changes in the expression and posttranslational modifications (phosphorylation, oxidation and nitrosylation) of ion channels and calcium transporters, respectively. PVBs lead to VT/VF especially in the presence of structural changes in the myocardium due to fibrosis and loss and hypertrophy of cardiomyocytes, leading to changes in the pathway and rate of impulse propagation^8,9^. However, the development of arrhythmia substrates in the heart and the underlying mechanisms are inadequately understood, especially in the elderly individuals and women. This is because the overwhelming majority of basic animal model studies devoted to identifying arrhythmia mechanisms have been conducted on male individuals, mostly at a young age, as highlighted by an extensive literature review recently published by our group^9^.

To alleviate the knowledge deficit in this area and to identify potential contributors to arrhythmia, we investigated the main components of the arrhythmia substrate (i.e. intracellular Ca^2+^ signaling, level of oxidative stress, electrophysiological parameters, fibrosis and cardiomyocyte hypertrophy) in the hearts of 3- and 24-month-old rats of both sexes and assessed their association with the incidence and severity of arrhythmias induced in ex vivo perfused hearts.

## Results

### Characterization of ex vivo induced arrhythmias in the hearts of young and old animals of both sexes

#### Premature ventricular beats and ventricular tachyarrhythmias

**Figure 1:**
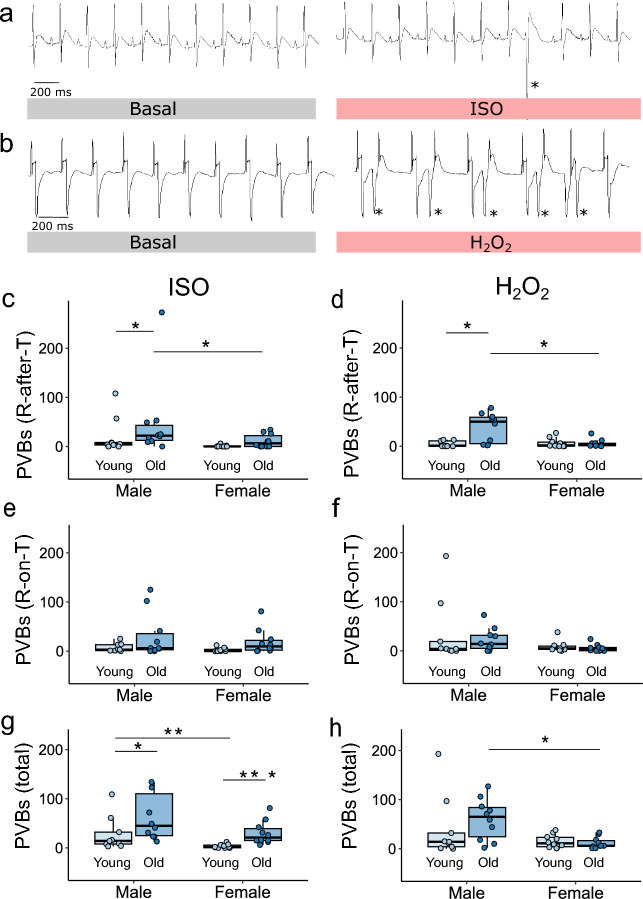
Efficacy of induction of premature ventricular beats in the rat heart ex vivo. (**a**) Examples of premature ventricular beats (PVBs) occurring after the end of the T-wave (R-after-T) and (**b**) during the T-wave (R-on-T) are marked with an asterisk. Numbers of R-after-T PVBs (**c**,**d**) and R-on-T PVBs (**e**,**f**) and their total number (**g**,**h**) were determined in the hearts of 3-month-old and 24-month-old male and female rats perfused ex vivo with isoproterenol (ISO) or hydrogen peroxide (H_2_O_2_), respectively. n = 9–10 hearts in each group. *p < 0.05, **p < 0.01, ***p < 0.001. Only statistically significant differences are shown.

**Figure 2:**
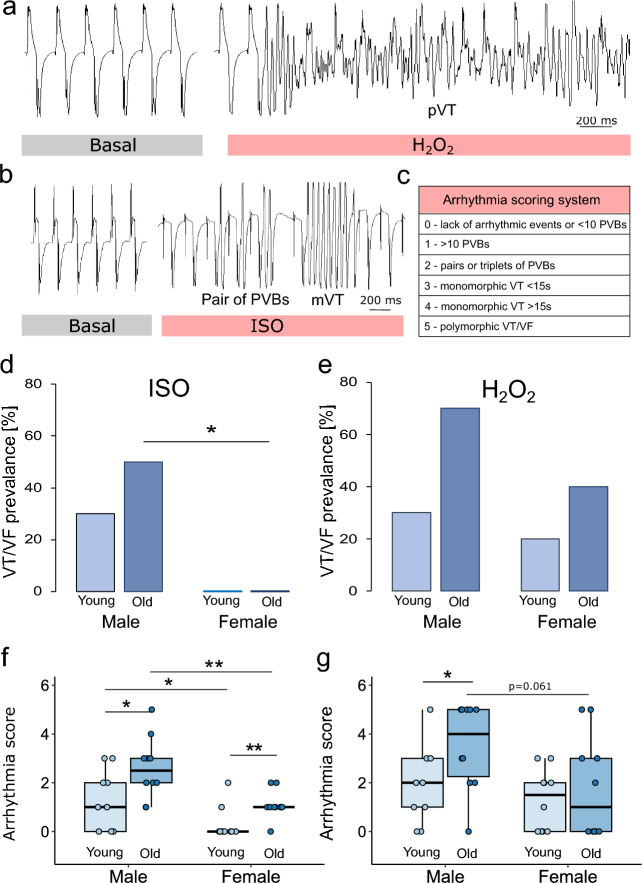
Efficacy of induction of ventricular tachycardia and ventricular fibrillation in the rat heart ex vivo. (**a**) Representative case of polymorphic ventricular tachycardia (pVT) induced by H_2_O_2_ in the heart of a 24-month-old male rat. (**b**) Representative case of monomorphic ventricular tachycardia (mVT) preceded by a pair of premature ventricular beats (PVBs) induced by ISO in the heart of a 24-month-old male rat. (**c**) Arrhythmia scoring system: points (0–5) were awarded for the number of PVBs, pairs or triplets of PVBs, short or long mVT and pVT progressing to VF, respectively. Percentage of hearts of 3-month-old and 24-month-old male and female rats with successful VT induction with isoproterenol (ISO) (**d**) or hydrogen peroxide (H_2_O_2_) (**e**). Arrhythmia scores assigned, based on the system shown in Table (**c**), to the hearts of 3-month-old and 24-month-old male and female rats after the administration of ISO (**f**) and H_2_O_2_ (**g**). n = 9–10 hearts in each group. *p < 0.05, **p < 0.01, ***p < 0.001. Only statistically significant differences are shown.

ECG recordings were analyzed manually by a blinded investigator for the occurrence of premature ventricular beats (PVBs) with separate consideration of PVBs occurring after the end of the T-wave (R-after-T) (Fig. 1a) and those occurring during its duration (R-on-T) (Fig. 1b), as well as for ventricular tachycardia (VT), both polymorphic (pVT) (Fig. 2a) and monomorphic (mVT) (Fig. 2b).

After ISO administration, the number of R-after-T PVBs was significantly greater in 24-month-old males than in females of the same age and in 3-months-old males. There was no difference in the number of R-after-T PVBs formed between young males and females or between young and old females (Fig. 1c). The addition of ISO to the perfusion solution also resulted in the appearance of numerous R-on-T PVBs. The highest number appeared in the elderly male group, but the difference between the groups was not significant (Fig. 1e). Like with ISO, the addition of H_2_O_2_ to the perfusion solution induced the highest number of R-after-T PVBs in elderly males, which was significantly greater than that in young males and females of the same age (Fig. 1d). In contrast, the induction of R-on-T PVBs was similarly effective in all study groups, but the highest number of R-on-T PVBs was detected in the group of old males (Fig. 1f). The total number of PVBs was sex- and age-dependent after ISO administration (Fig. 1g), while H_2_O_2_ induced more PVBs in old males than in females of the same age (Fig. 1h).

Under ISO perfusion VTs appeared in 50% of 24-month-old males and in 30% of 3-month-old males while they did not arise in the heart of any female regardless of age. Statistical analysis revealed that the incidence of VT was significantly greater in 24-month-old males than in age-matched females (Fig. 2d). On the other hand, H_2_O_2_ perfusion induced VTs in the hearts of all the study groups, most frequently in old males (in 70% of the individuals) and least frequently in young females (in 20%). However, the differences between the groups did not reach statistical significance (Fig. 2e).

#### Arrhythmia score

Arrhythmia severity was estimated using an arrhythmia score calculated according to the scale shown in Fig. 2c. For ISO-induced arrhythmias, the arrhythmia score was highest in old males and was significantly greater than that in young males and old females. Young females had a lower incidence of arrhythmia than young males and old females did (Fig. 2f).

The arrhythmia severity was greater after H_2_O_2_ administration than after ISO administration. A maximum score (5 points) was obtained for 8 hearts compared to one heart after ISO (p = 0.029). The arrhythmia severity was greater in the hearts of males aged 24 months than in those aged 3 months. A clear trend (p = 0.061) toward higher arrhythmia scores was also observed in older males than in females of the same age (Fig. 2g).

### Arrhythmic substrate in the hearts of young and old animals of both sexes

#### Electrophysiological parameters

**Figure 3:**
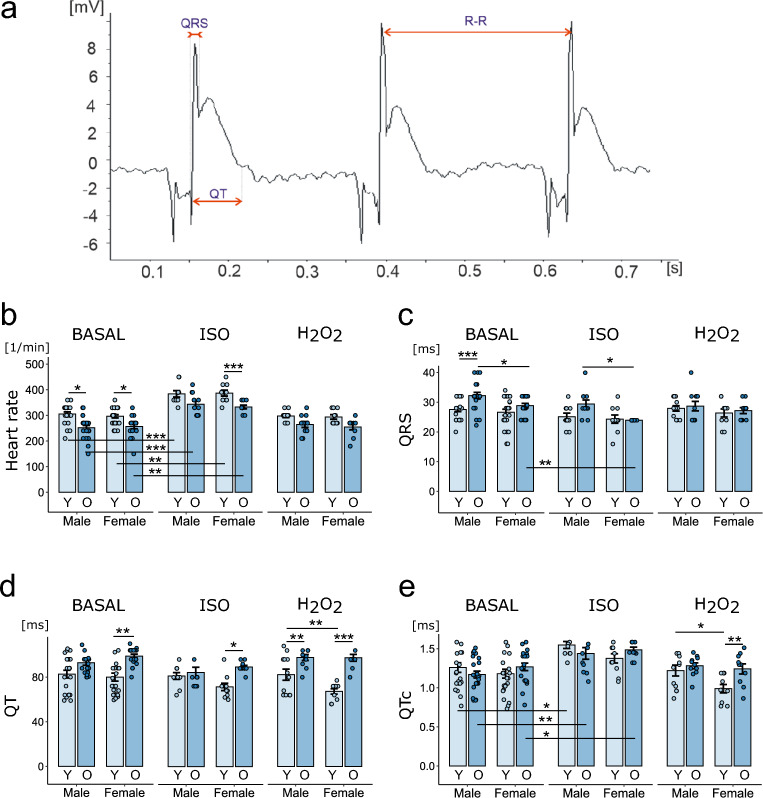
Electrophysiological parameters of ex vivo hearts before and after arrhythmia induction. (**a**) Representative recording of the ECG signal from the surface of the rat heart ex vivo. Heart rate (HR) (**b**), QRS complex duration (**c**), QT interval duration (**d**) and corrected QT interval duration (**e**) in hearts of 3-month-old and 24-month-old male and female rats measured before (basal) and after 20 min of perfusion with isoproterenol (ISO) and hydrogen peroxide (H_2_O_2_). HR and QRS and QT durations were calculated as the mean values from five consecutive beats. n = 9–10 hearts in each group. *p < 0.05, **p < 0.01, ***p < 0.001. Only statistically significant differences are shown (ISO and H_2_O_2_ vs. basal).

The intrinsic heart rate (HRi), duration of QRS complex and QT interval were measured manually (Fig. 3a) in ECG recordings before and after the administration of ISO and H_2_O_2_. The HRi before inducer administration (baseline) was similar in both sexes, regardless of age, and was lower in old animals than in young animals (Fig. 3b). The duration of QRS complex was significantly longer in males aged 24 months than in females of the same age and in males aged 3 months (Fig. 3c). The QT interval was longest in older females, but a statistically significant difference was detected only between the older and young female groups (Fig. 3d). We did not detect differences in the corrected QT (QTc) interval between the groups (Fig. 3e).

Under ISO perfusion, the HRi increased in all study groups indicating the preservation of chronotropic reserve during the aging process in rats. Under baseline conditions, there were no differences in HRi between males and females. In contrast, older age resulted in a decrease in HRi. H_2_O_2_ had no significant effect on the HRi and, after H_2_O_2_ administration, the HRi was similar in all study groups (Fig. 3b). Administration of ISO increased the conduction velocity (shorter QRS) compared to that measured under basal conditions in all study groups, but this change reached statistical significance only in the group of females at 24 months. The duration of QRS complex in ISO-perfused hearts was significantly longer for older males than for females of the same age. Interestingly, H_2_O_2_ perfusion abolished the differences in QRS duration between the groups by shortening QRS duration in the group of older males (Fig. 3c). Neither ISO nor H_2_O_2_ perfusion significantly affected QT duration in either group compared to that in the basal conditions. During ISO perfusion, the QT duration, as under basal conditions, was longer in old females than in young females. In contrast, H_2_O_2_ perfusion led to differences in QT duration between groups. QT was longer in old animals than in young animals regardless of sex, and was longer in young males than in young females (Fig. 3d). QTc in ISO-perfused hearts was comparable in all groups and significantly longer than that under basal conditions (in the young female group, the difference did not reach statistical significance due to the increase in HRi). In H_2_O_2_-perfused hearts, the QTc was not significantly different from that calculated under basal conditions and was significantly longer in young males than in females of the same age and was longer in old females than in young females (Fig. 3e).

#### Ca^2+^ signaling

**Figure 4:**
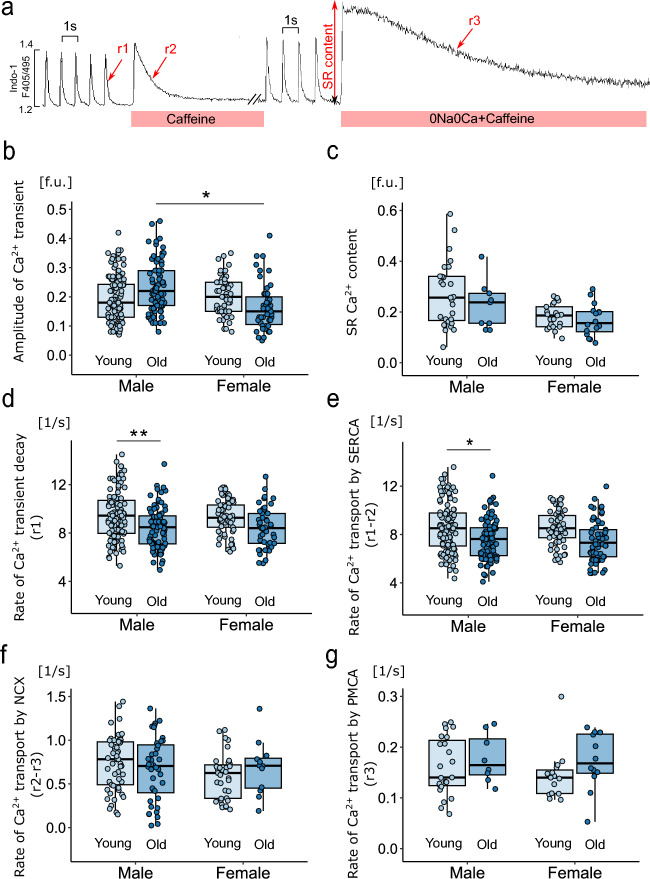
Parameters of intracellular Ca^2+^ signaling in isolated cardiomyocytes. (**a**) Experimental protocol: cardiomyocytes were stimulated at 1 Hz. Caffeine was administered to cardiomyocytes perfused with Tyrode's solution (TS) or Na^+^- and Ca^2+^-free solution (0Na0Ca). Exponential curves were fitted to the decaying part of electrically and caffeine-evoked Ca^2+^ transients, and the rate constants of their decay (r1, r2 and r3) were calculated. The rates of Ca^2+^ transport by sarcoplasmic reticulum Ca^2+^-ATPase (SERCA2a), the Na^+^/Ca^2+^ exchanger (NCX1) and the sarcolemmal Ca^2+^-ATPase (PMCA) were calculated according to the formulas r_SERCA_ = r1–r2, r_NCX_ = r2–r3 and r_PMCA_ = r3, respectively. The amplitude of Ca^2+^ transients evoked by caffeine in cardiomyocytes perfused with 0Na0Ca solution was taken as a measure of sarcoplasmic reticulum (SR) Ca^2+^ content. (**b**) Amplitude of Ca^2+^ transients. (**c**) SR Ca^2+^ content. (**d**) The rate of Ca^2+^ transient decay. The rate of Ca^2+^ transport by SERCA2a (**e**), NCX1 (**f**) and PMCA (**g**). Electrically evoked Ca^2+^ transients (**b**,**d**,**e**) were analyzed in 64–121 cardiomyocytes; caffeine-evoked Ca^2+^ transients under perfusion with Tyrode’s solution (**f**) in 22–53 cardiomyocytes; and caffeine-evoked Ca^2+^ transients under perfusion with 0Na0Ca solution (**c**,**g**) in 8–27 cardiomyocytes isolated from 4–5 hearts in each group. *p < 0.05; **p < 0.01; ***p < 0.001. Only statistically significant differences are shown.

**Figure 5:**
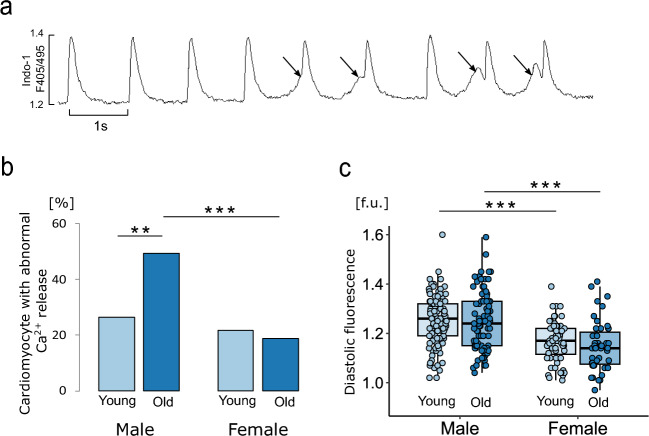
Spontaneous ryanodine receptor activity and diastolic Ca^2+^ concentration. (**a**) Example recording of Ca^2+^ transients electrically stimulated at 1 Hz with visible spontaneous Ca^2+^ release from the sarcoplasmic reticulum manifested as aftertransients indicated by arrows. (**b**) Relative number of cardiomyocytes with abnormal Ca^2+^ release analyzed in 120 s recordings for each cardiomyocyte studied. (**c**) Diastolic fluorescence measured as an index of the diastolic Ca^2+^ concentration. n = 64–121 cardiomyocytes isolated from 4–5 hearts in each group. *p < 0.05; **p < 0.01; ***p < 0.001. Only statistically significant differences are shown.

The protocol for measuring Ca^2+^ transient parameters, the SR Ca^2+^ content and the activity of Ca^2+^ transporters is shown in Fig. 4a. The amplitude of Ca^2+^ transients was significantly greater in old males than in females, while SR Ca^2+^ content was not significantly different between the groups (Fig. 4b,c). This may suggest that the fractional SR release is greater in the old male group than in the old female group due to the greater sensitivity of RyRs to diastolic Ca^2+^ and the greater tendency for spontaneous opening of RyRs, which are the cause of PVBs. Indeed, the percentage of cardiomyocytes in which there were spontaneous openings of RyRs manifested by the appearance of so-called aftertransients (Fig. 5a) was almost twice as high in the group of 24-month-old males as in females in the same age group and in the group of 3-month-old males (Fig. 5b). The diastolic Ca^2+^ concentration was greater in males than in females, regardless of age (Fig. 5c).

The rate of Ca^2+^ transient decay was lower in old animals than in young animals, but a statistically significant difference was observed only between the old and young male groups (Fig. 4d). This difference was due to a decrease in SERCA2a activity in the old male group compared to the young male group (Fig. 4e). There were no differences in the activity of NCX1 or the PMCA between the study groups (Fig. 4f,g).

#### Fibrosis and cardiomyocyte hypertrophy

**Figure 6:**
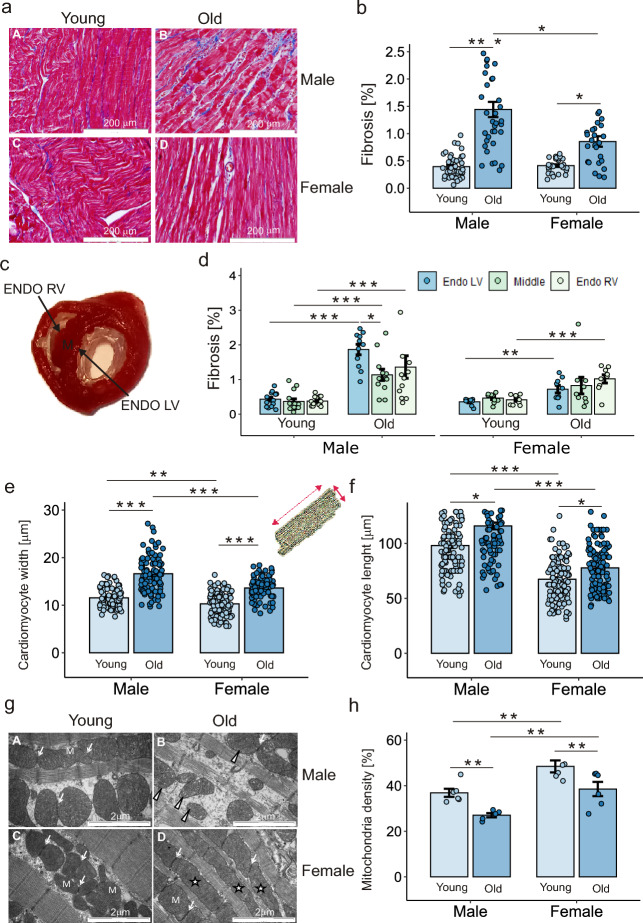
Structural remodeling of the myocardium: fibrosis, cardiomyocyte hypertrophy and mitochondrial density. (**a**) Representative sections of the ventricular septum from the heart of 3-month-old male and female (**A**,**C**) and 24-month-old male and female (**B**,**D**) rats stained with Masson’s trichrome stain. Magnification × 40. Blue staining indicates areas of myocardial fibrosis. (**b**) Fibrosis across the ventricular septum according to the age and sex of the rats. (**c**) Cross-section of the heart with visible myocardial layers: subendocardial layer of the left (Endo LV) and right (Endo RV) ventricles and the middle (M) layer of the ventricular septum. (**d**) Fibrosis evaluated separately in the myocardial layers of the Endo LV, Endo RV and M. Width (**e**) and length (**f**) of cardiomyocytes (n = 100–120) isolated from 4–5 hearts in each group (**g**) Representative area with mitochondria in cardiomyocyte sections according to age and sex: 3-month-old male and female (**A**,**C**) and 24-month-old male and female (**B**,**D**) rats. Normal mitochondria (M); mitochondria with a normal ultrastructure but double or triple in length (white star); contact between the outer membranes of adjacent mitochondria (fine white arrow); and abnormal mitochondria with distended and irregularly arranged mitochondrial cristae (white arrowhead). (**h**) Mitochondrial density in cardiomyocytes according to the age and sex of the rats. n = 3–5 hearts in each group for (**b**), (**d**) and (**h**). *p < 0.05; **p < 0.01; ***p < 0.001. Only statistically significant differences are shown.

Fibrosis of the interventricular septum was similar in young animals of both sexes, whereas in older animals was significantly greater in males than in females (Fig. 6a,b). Analysis of fibrosis separately in the three muscle layers of the septum, the subendocardial layer of the left ventricle (ENDO LV), the middle layer (M) and the subendocardial layer of the right ventricle (ENDO RV) (Fig. 6c) revealed comparable phenomena in all layers in young animals of both sexes. With age, fibrosis in males increased significantly in all 3 layers but was most severe in the ENDO LV. This led to heterogeneous fibrosis throughout the heart muscle, which provides a structural substrate for arrhythmias (Fig. 6d, left panel). In females, fibrosis also increased with age, but the process was less severe than that in males. Moreover, in old females, we detected no difference in the degree of septal fibrosis in the different muscle layers of the interventricular septum (Fig. 6d, right panel).

The increase in fibrosis in older animals was accompanied by cardiomyocyte hypertrophy, which was defined by an increase in cardiomyocyte length and width. Cardiomyocyte hypertrophy was significantly greater in older males than in females of the same age (Fig. 6e,f).

#### Oxidative stress and the antioxidant defense system

The main sources of superoxide anion radical (O_2_^**·**−^) production in the heart are mitochondria and NADPH oxidases (NOXs). Ultrastructural analysis of cardiomyocytes revealed a decrease in mitochondrial density with age in both sexes, but regardless of age, this change was higher in females than in males (Fig. 6h). In addition, differences in the structure, size and cellular localization of mitochondria were noticeable with respect to age and sex (Fig. 6g). Mitochondria in the cardiomyocytes of young animals were arranged in single or double rows. Their characteristic features included an oval or round shape, varying size and parallel mitochondrial cristae embedded in a uniformly electron-dense matrix (Fig. 6g, panels A and C). In contrast, 24-month-old rats exhibited changes in mitochondrial arrangement and morphology (Fig. 6g, panels B and D). In old females, some mitochondria were larger and longer than those present in young individuals. The latter phenomenon may be a consequence of mitochondrial fusion. Both changes help compensate for the decreased metabolic efficiency of mitochondria. The structure of the matrix was normal with properly arranged cristae. In some cardiomyocytes, individual lipid droplets and/or autophagosomes were visible near the mitochondria (Fig. 6g, panel D). In contrast, in 24-month-old males, the mitochondria were polymorphic and variable in size and were rarely arranged in bundles. Some of the mitochondria had abnormal inner membrane structures that formed irregular cristae, which may be associated with impaired mitochondrial bioenergetic function (Fig. 6g, panel B).

**Figure 7:**
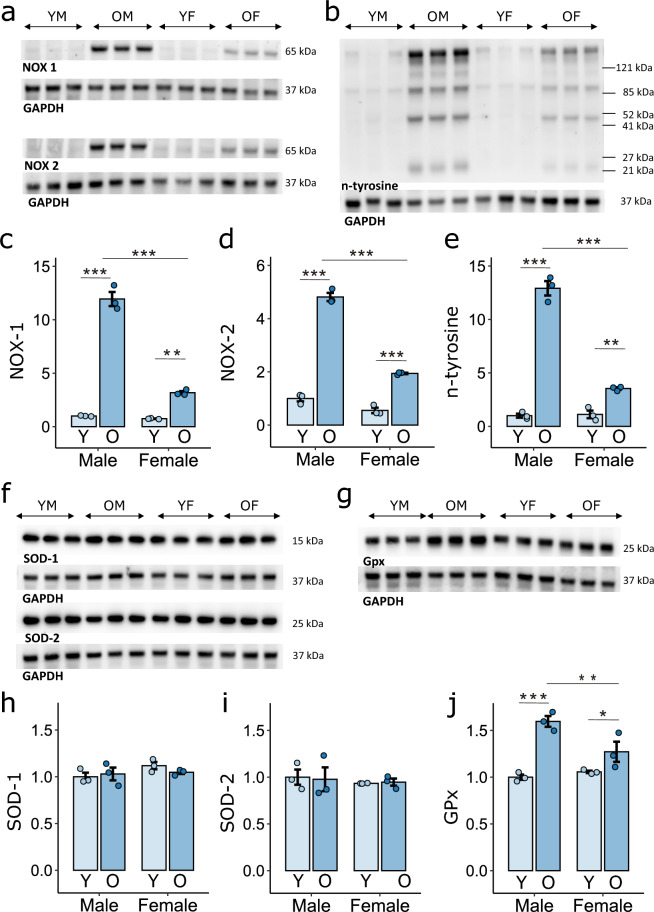
Oxidative stress and antioxidant defense. Expression of NADPH oxidase-1 (NOX-1) (**a,c**), NADPH oxidase-2 (NOX-2) (**a**,**d**), nitrated tyrosine (n-tyrosine) (**b**,**e**), superoxide dismutase-1 (SOD-1) (**f**,**h**), superoxide dismutase-2 (SOD-2) (**f**,**i**), and glutathione peroxidase (GPx) (**g**,**j**) quantified by western blot analysis in homogenates of 3-month-old and 24-month-old male and female rat hearts. n = 3 hearts in each group. *p < 0.05; **p < 0.01; ***p < 0.001. YM, OM, YF, and OF—young and old males and females, respectively. Only statistically significant differences are shown.

In addition, the expression of NOX-1 and NOX-2, the main sources of O_2_^**·**−^ in the vasculature, was more than 10 times greater in the hearts of old males than in those of young individuals. We also noted a marked increase in the expression of these genes in old females but the level was much lower than that in males (Fig. 7a,c,d). Consistent with these observations, the presence of 3-nitrotyrosine, a marker of oxidative protein damage, was low in groups of young animals of both sexes and increased significantly with age. This increase was several times greater in the hearts of old males than in those of females (Fig. 7b,e).

In contrast to the strong increase in NOX-1 and NOX-2 expression with age, which was particularly pronounced in males, the expression of SOD-1 and SOD-2, which inactivate O_2_^**·**−^ by converting it to H_2_O_2_, was comparable in the hearts of young and old animals of both sexes (Fig. 7f,h,i). On the other hand, the expression of glutathione peroxidase (GPx), which in turn inactivates H_2_O_2_ by breaking it down to H_2_O, increased with age, especially in males (Fig. 7g,j).

## Discussion

To our knowledge, this is the first study comparing the susceptibility of hearts from young and old rats of both sexes to ex vivo induction of arrhythmias by inducers that mimic sympathetic nervous system activation (ISO) or oxidative stress (H_2_O_2_), both of which accompany cardiovascular disease. We showed that after ISO administration, VT/VFs were formed only in the hearts of males, especially in those of older animals. In contrast, H_2_O_2_ induced the formation of VT/VFs in the hearts of rats of both sexes, but more frequently in older males. In addition, we identified several components of the arrhythmia substrate that develop in the myocardium during the aging process, including (1) high spontaneous RyR activity, (2) fibrosis of varying severity in different layers of the myocardium (nonheterogenic fibrosis), and (3) high levels of oxidative stress as measured by nitrated protein tyrosine levels. All of these elements develop during the aging process at a much greater intensity in the hearts of male individuals. On the other hand, in the hearts of aging females, antioxidant defense at the level of H_2_O_2_ detoxification, measured as glutathione peroxidase expression, was weaker than that in males of the same age.

Life-threatening ventricular arrhythmias such as VT and VF most often arise by a re-entry mechanism. A re-entry circuit is established when a triggering factor, most often a premature ventricular beat (PVB), coexists with favorable electrophysiological substrate such as dispersion of repolarization or slow rate of impulse propagation. The main inducers of PVBs are early-type (EADs) or late-type (DADs) afterdepolarizations, occurring during or after the AP, respectively. The source of DADs is transient inward current (I_ti_) generated mainly by NCX1, activated by Ca^2+^ released spontaneously from the SR. EADs, especially those occurring at the end of the repolarization phase, can also be a consequence of I_ti_ activation but mainly occur as a result of spontaneous reopening of Na^+^ or Ca^2+^ channels favored by AP elongation. Hence, disturbances of intracellular Ca^2+^ signaling and AP elongation are factors that favor PVBs and increase the risk of VT/VF^8,10^.

Spontaneous opening of RyRs and activation of I_ti_ can occur as a result of SR Ca^2+^ overload, an increase in diastolic Ca^2+^ concentration, and/or increased RyR sensitivity to Ca^2+^ ions. The latter phenomenon is a consequence of posttranslational modifications of RyRs, such as phosphorylation and oxidation^11^. The main kinases responsible for the phosphorylation of RyRs are protein kinase type A (PKA) and calcium and calmodulin-dependent kinase (CAMKII), whose activation occurs in direct or indirect response to stimulation of β-adrenergic receptors, respectively^12^. A potent modifier of the functional state of RyRs is also the oxidation of cysteine residues under conditions of increased oxidative stress, which leads to permanent modification of RyRs through the formation of disulfide bridges and spontaneous Ca^2+^ leakage from the SR. Hence, the two arrhythmia inducers used in this study may themselves modify the functional state of RyRs and/or exacerbate posttranslational modifications of RyRs present prior to their administration. Indeed, in the ex vivo perfused heart model, both ISO and H_2_O_2_ led to the formation of multiple PVBs. The number of PVBs and their effectiveness in inducing VT/VF differed significantly between groups of rats, suggesting the existence of a different arrhythmic substrate in the hearts of young and old animals of both sexes. To determine the nature of this substrate, we investigated disturbances in Ca^2+^ signaling in isolated cardiomyocytes with a particular focus on spontaneous Ca^2+^ release through RyRs; parameters of oxidative stress and the antioxidant defense system; QT and QRS durations, giving insight into AP duration and ventricular conduction rate, respectively; and the degree of myocardial fibrosis and cardiomyocyte hypertrophy, which modify the rate of impulse propagation.

We showed that the main change occurring in the aging process at the level of intracellular Ca^2+^ signaling is a decrease in the rate of Ca^2+^ transient decay in the cardiomyocytes of old animals resulting from a decrease in SERCA2a transport activity and preserved Ca^2+^ efflux by NCX1 and PMCA. Changes in SERCA2a activity were evident during the aging process in both sexes but reached statistical significance only in the hearts of old males. Consistent with our findings, it has been shown that in aged male SERCA2a expression decreases at both the mRNA^13^ and protein^14^ levels, while SERCA2a expression at the protein level in old female rats remains at a level similar to that in young animals^15^. Additionally, in cardiomyocytes isolated from female rabbit hearts, SERCA2a transport activity was not significantly altered by aging^16^. Like in the current study, in our previous work, SERCA2a activity was significantly reduced in elderly males subjected to 6 h of ischemia but was preserved in females of the same age^17^.

In addition, we observed a marked age- and sex-dependent difference in the spontaneous activity of RyRs in electrically stimulated cardiomyocytes. This was manifested by the presence of ‘aftertransients’ resulting from the spontaneous opening of RyRs independent of synchronized Ca^2+^ release from the SR due to Ca^2+^ influx through L-type calcium channels. The hyperreactivity of RyRs was significantly greater in males than in females, and the difference increased dramatically with age. Other authors have also studied spontaneous RyR activity but in resting cardiomyocytes, monitoring the frequency of so-called ‘calcium sparks’^18^. As in our study, spontaneous RyR activity increased with age in rats of both sexes and in male mice^19–21^. In contrast, in female rabbits, the frequency of ‘calcium sparks’ was similar between young and old individuals^16^. On the other hand, in young rats, the frequency of calcium sparks was similar in both sexes, but their amplitude and duration were significantly lower in females, which may indicate greater spontaneous Ca^2+^ leakage from the SR in males^19^. To our knowledge, no work to date has directly compared either the incidence of ‘calcium sparks’ in resting cardiomyocytes or the spontaneous opening of RyRs in working cardiomyocytes in young and old rats of both sexes.

The increase in the spontaneous activity of RyRs in the cardiomyocytes of old males may have been partly due to the increase in diastolic Ca^2+^ concentration in this group. However, this does not appear to be the main reason, as spontaneous RyR activity is almost twice as high in old males as in young males at comparable diastolic Ca^2+^ concentrations in these two groups of animals. These results suggest that the high spontaneous activity of RyRs in old males was mainly due to their increased sensitivity to Ca^2+^ ions. However, the results of previous studies suggest that this is not due to the higher level of RyR phosphorylation by PKA and/or CaMKII. CaMKII activity decreases with age in males, and the level of phosphorylation of RyRs, as well as other proteins such as SERCA2a and phospholamban (PLB), by this kinase is reduced in old male individuals^22^. In addition, the levels of intracellular cAMP, which activates PKA, decrease in males during the aging process^23^. These observations suggest that the modifications of RyRs induced by reactive oxygen species underlie the spontaneous opening of RyRs in old males. Indeed, studies have shown that oxidation of thiol (-SH) groups is an irreversible modifier of the functional state of RyRs and leads to increased SR Ca^2+^ leakage and exacerbation of ventricular arrhythmias^24–26^. In addition, Cooper et al. showed that inactivating mitochondrial-derived ROS reduces RyR hyperresponsiveness, SR Ca^2+^ leakage, and proarrhythmic calcium waves in elderly rabbit hearts^16^.

In fact, in our study, the enhanced spontaneous RyR activity in old males was accompanied by high levels of oxidative stress. In this group of animals, the level of nitrated tyrosine, a marker of oxidative stress, was several times greater than that in the group of even-aged females and more than 10 times greater than that in young animals of both sexes. In the heart muscle, O2^**·**−^ is produced mainly in mitochondria as a byproduct of aerobic respiration and by NOX-1 and NOX-2, enzymes highly expressed in the vasculature and cardiomyocytes^26,27^. While the expression of NOX-1 and NOX-2 was several times greater in the heart tissue of old males than in that of the other groups of animals, we noted a decrease in mitochondrial density in both sexes during the aging process. However, in the hearts of old males, abnormal mitochondrial inner membrane structures were visible in microscopic images, which may lead to uncoupling of the respiratory chain and increased total O2^**·**−^ production despite the decrease in mitochondrial density^28–30^. On the other hand, a reduction in mitochondrial density can lead to an energy deficit in cardiomyocytes, one symptom of which can be reduced activity of SERCA2a, a Ca^2+^ transporter that consumes significant amounts of cellular ATP. In old females, despite the decrease in density, the mitochondria presented a normal structure. These mitochondria were also longer than the mitochondria of young individuals, which may be the result of their fusion, a phenomenon that allows them to compensate for the weaker metabolic efficiency of mitochondria. Moreover, elongated mitochondria have been shown to correlate with more efficient ATP production^31^. This difference may explain why the change in SERCA2a transport activity was less severe during aging in females than in males. In addition, the presence of autophagosomes near mitochondria in the hearts of 24-month-old females may indicate the selective sequestration of damaged mitochondria for degradation and removal from the cell. This may reduce O2^**·**−^ production by damaged mitochondria and determine the lower level of oxidative stress in 24-month-old females than in males of the same age^29,30^.

Despite strongly increased O2^**·**−^ production in the hearts of 24-month-old males, antioxidant defense at the level of O2^**·**−^ detoxification was not enhanced. The expression of SOD-1 and SOD-2, which transform O2^**·**−^ to H_2_O_2,_ was the same as that in young animals. In contrast, the expression of GPx, which converts H_2_O_2_ to H_2_O, significantly increased during the aging process in males, and this increase was more pronounced than that in females. However, this phenomenon failed to compensate for the increased production of ROS in old males, which leads to direct or indirect modification of proteins by oxidation of thiol groups or tyrosine nitration, respectively. On the other hand, the lower expression of GPx in elderly females than in males of the same age may explain the occurrence of VT/VF in almost half of the 24-month-old females after H_2_O_2_ administration, despite a significantly less developed arrhythmic substrate than in males: lower spontaneous RyR activity and less severe structural changes in the myocardium (see below). It seems that females, due to lower antioxidant defense at the level of H_2_O_2_, have a much higher risk of arrhythmia after H_2_O_2_ perfusion than after ISO stimulation, resulting in no VT/VF induction.

Other studies have revealed greater oxidative stress in males and sex-dependent differences in the antioxidative defense system, but comparative studies have been conducted only in young animals^32,33^. Barp et al. showed that the severity of oxidative stress as measured by lipid peroxidation, was significantly greater in male rats than in female rats, but only young animals at 9 weeks of age were included in the study^34^. O2^**·**−^ production in isolated rat aortas under basal conditions, indicating NOX activity, was significantly higher in males than in females, but a study was also conducted on young animals^35^. They also found that SOD activity measured in myocardial homogenates was lower in young males than in females, while GPx activity was greater^34^. Moreover, Chen et al. reported greater GPx activity in the hearts of male mice than in those of female mice, but the age of the animals was not considered in their study. Most likely, the decreased GPx activity in females is compensated by estrogen, which acts as an antioxidant due to the presence of a phenolic hydroxyl group^36^. It is likely that the increase in GPx expression in females during the aging process, which we show here, is a response to the aging-induced decrease in estrogen levels^37^.

The increase in oxidative stress in old males suggested that the oxidation of thiol groups and/or nitration of tyrosine residues in RyRs may be the main cause of increased spontaneous RyR activity and subsequent PVBs. This basal overreactivity of RyRs in the hearts of old males was further enhanced by the agents used to induce arrhythmias ex vivo, which exacerbated oxidative stress (H_2_O_2_) or introduced additional modifications of RyRs through their phosphorylation (ISO). In addition to the alteration of RyR function by aggravated oxidative stress, SERCA2a function may be also impaired by the oxidation of thiol groups and tyrosine nitration^26,38^, which may explain reduction in SERCA2a activity and the decrease in the rate of Ca^2+^ transient decay in our old males.

Another factor, in addition to impaired Ca^2+^ signaling, which favors the formation of PVBs, may be the prolongation of AP, which promotes formation of EADs. However, under ex vivo conditions, the AP duration appears to have a negligible effect on the incidence of PVBs. QT duration, an indirect measure of AP duration, was greatest in older females. However, this did not result in a higher incidence of PVBs, in this group of animals. The prolongation of the QT interval was due to a slowing of the HRi in older animals, as the QTc was comparable in all study groups. A decrease in HRi during the aging process was also found in our earlier study. We showed that it was due to a decrease in the expression of genes encoding proteins responsible for resting depolarization in sinus node cells^39^.

The effectiveness of PVBs in inducing re-entrant arrhythmias depends on the presence of an arrhythmic substrate in the myocardium, such as increased fibrosis or cardiomyocyte hypertrophy, which reduce the impulse propagation rate. Indeed, in the elderly males in whom we found the most VT/VF, the ventricular conduction rate, as measured by QRS duration, was significantly lower. This findings is consistent with the significantly greater degree of fibrosis and more severe cardiomyocyte hypertrophy in the group of old males than in the group of females of the same age.

Furthermore, by examining fibrosis separately in individual layers of the myocardium in the interventricular septum, we showed that while in young animals, the degree of fibrosis is comparable throughout the tissue, with aging, there appears to be an uneven increase in fibrosis in individual layers of muscle, with the greatest fibrosis occurring in the subendocardial layer of the left ventricle. We observed this interesting phenomenon only in males. A varying degree of fibrosis in different areas of the myocardium in old rats was also demonstrated by Morita et al., but only males were included in the study^40^. Differences in the level of fibrosis at the border of individual layers of myocardium create an additional arrhythmic substrate. In model studies, it has been shown that when PVBs arise in tissue with a lower degree of fibrosis, they are efficiently transmitted to adjacent areas with a higher degree of fibrosis^41^.

## Conclusions

We have shown that sex strongly influences the development of an arrhythmia substrate during aging. This substrate determines the occurrence of life-threatening arrhythmias in the presence of additional stimuli with proarrhythmic potential, such as catecholamine stimulation or increased oxidative stress, which accompany most cardiovascular diseases. We have shown that increased spontaneous RyR activity, increased oxidative stress and uneven fibrosis in different layers of the myocardium correlate with increased arrhythmic susceptibility and develop to a much greater extent in aging male hearts than in female hearts.

Moreover, our results also suggest that male sex is associated with increased susceptibility to catecholamine-induced arrhythmias and point to the validity of interventions to reduce the effects of excessive catecholamine stimulation in male individuals. In turn, oxidative stress increases the arrhythmic potential in both sexes. In addition, older females, due to their lower antioxidant potential than males, may be particularly susceptible to arrhythmias in diseases accompanied by increased oxidative stress, such as acute ischemia, heart failure or diabetes.

## Methods

Male and female Wistar rats (n = 73) aged 3 and 24 months were used in the study in compliance with local and institutional regulations. The study conforms to the Guide for the Care and Use of Laboratory Animals, US National Institutes of Health (NIH Publication No. 85-23, revised 1996) and was approved by the local Ethics Committee (Second Warsaw Local Ethics Committee for Animal Experimentation). The authors complied with the Animal Research: Reporting of In Vivo Experiments (ARRIVE) guidelines.

### Experimental design

Rats were anesthetized by intraperitoneal administration of ketamine (100 mg/kg body weight) and xylazine (5 mg/kg body weight). The hearts taken from young (3 months old) and old (24 months old) female and male rats were randomly assigned to subgroups (n = 9–10 hearts in each) for arrhythmia induction with isoproterenol (ISO) or hydrogen peroxide (H_2_O_2_) and mounted in the Langendorff system. Two platinum electrodes were implanted into the heart muscle to obtain ECG recordings. After a 10-min stabilization period, the hearts were perfused for 20 min with Krebs–Henseleit solution containing H_2_O_2_ (0.2 mM) or ISO (0.1 µM) to induce arrhythmia. The ECG signals were then manually analyzed off-line by a blinded investigator. Additionally, hearts from 7 to 10 rats in each group were used to assess the arrhythmic substrate. Four to five hearts from each group were subjected to enzymatic digestion to isolate cardiomyocytes, assess their dimensions and characterize intracellular Ca^2+^ signaling. Tissue was collected from the remaining three to five hearts from each group for histological and biochemical analyses to determine myocardial fibrosis, mitochondrial density and structure, and to measure oxidative stress and antioxidant enzyme expression.

### Evaluation of ventricular arrhythmias

Two platinum electrodes were inserted into the myocardium to a depth of approximately 1 mm, one in the apex and one in the left atrial appendage, and connected to an ECG telemetry transmitter (*Data Sciences International, St. Paul, MN*). A receiver was placed next to the perfused heart to continuously capture the ECG signal. The signals were stored on a computer disk and analyzed manually offline (*ART 4.1 Gold Acquisition & Analysis Software, Data Sciences*).

The intrinsic heart rate (HRi), QRS complex and QT interval durations were measured before and after arrhythmia induction as average values from five consecutive beats. The duration of the QT interval was corrected according to Fridericia’s formula (*QT*_*c*_ = $\frac{QT}{R{R}^{1/3}}$). After the addition of arrhythmia inductors, the number of premature ventricular beats (PVBs), the number of PVB pairs and triplets and the number of episodes of ventricular tachycardia (VT) and ventricular fibrillation (VF) were analyzed. Ventricular arrhythmias were classified according to the Lambeth Conventions guidelines referring to animal studies^42^. The PVBs were defined as beats with a morphology distinct from that of a normal sinus beat within the T-wave (R-on-T) (Fig. 1b) or after its completion (R-after-T) (Fig. 1a). Monomorphic ventricular tachycardia (mVT) was defined as at least 4 consecutive premature ventricular beats of constant morphology, amplitude, duration and frequency (Fig. 2b), while polymorphic ventricular tachycardia (pVT) was defined as at least 4 consecutive premature ventricular beats of variable amplitude, duration or frequency, of which at least one parameter change was progressive (Fig. 2a). VF was defined as 4 consecutive premature ventricular beats of variable amplitude, duration or frequency, for which the change in any parameter was not progressive. Arrhythmia severity was described by the number of points (arrhythmia score) assigned to each arrhythmic episode according to the Lambeth Conventions^42^ (Fig. 2c).

### Calcium signaling in isolated cardiomyocytes and cardiomyocyte dimensions

Left ventricular cardiomyocytes were isolated according to standard procedures^43^, incubated for 15 min with 10 mM Indo-1 acetoxymethyl ester (*Sigma*) and superfused at 37 °C with Tyrode’s solution containing 1.8 mmol/l Ca^2+^. The difference between the systolic and diastolic Indo-1 fluorescence (excited at 365 nm and measured as the ratio of fluorescence at 405 and 495 nm) was used as a measure of the amplitude of Ca^2+^ transients.

The rate of Ca^2+^ transport by sarcoplasmic reticulum (SR) Ca^2+^-ATPase (SERCA2a), the Na^+^/Ca^2+^ exchanger (NCX1) and the plasma membrane Ca^2+^-ATPase (PMCA) was estimated from the rate constants (r1, r2 and r3) of the single exponential curves fitted to electrical and caffeine-evoked Ca^2+^ transient decay, as presented in Fig. 4a. The rate constants of Ca^2+^ transient decay for SERCA2a and NCX1 were calculated according to the formulas r_SERCA_ = r1–r2 and r_NCX_ = r2–r3, respectively, while r3 was taken as the measure of the rate of Ca^2+^ transport by the PMCA (r3 = r_PMCA_). The SR Ca^2+^ content was estimated from the amplitude of caffeine-evoked Ca^2+^ transients in myocytes superfused with Na^+^ and Ca^2+^-free (0Na0Ca) solution^44^.

### Myocardial fibrosis

Hearts were excised immediately after thoracic opening. The septum of each heart was fixed in 10% buffered formalin (pH 7.4) and processed for paraffin blocks according to the standard histological procedures. Sections of the septum (5 μm thick), were deparaffinized and stained with Masson’s trichrome stain. Digital images of the septum with no visible microvessels were taken by a light microscope (BX-10, *Olympus*) at 40 × objective magnification. Myocardial fibrosis was measured on images taken from 3 to 5 hearts in each group for the whole interventricular septum and separately for the subendocardial layer of the left and right ventricles and the middle layer of the septum. Nine images (3 in each layer) were analyzed for each heart. The blue-stained areas seen on the images were assessed with CellSense morphometric software (*Olympus*), which automatically defined the results as the percentage of an analyzed field.

### Mitochondrial ultrastructure and density

Small specimens of myocardium obtained from the LV were excised by fixation in 2.5% glutaraldehyde in phosphate buffer (pH 7.4), postfixed in 1% buffered osmium tetroxide, dehydrated in solution with ascending alcohol concentrations and processed for Epon embedding. Ultrathin sections were cut from areas containing longitudinal sections of cardiomyocytes in the middle layer of the left ventricle, contrasted with uranyl acetate and lead citrate, and examined by TEM (*Jeol JEM 1011, Japan*). Ultrastructural details of cardiomyocyte mitochondria were collected on images at 10,000 ×, 20,000 × and 40,000 × magnification. Five to seven LV samples were obtained from 3 to 5 hearts in each group. Ten images taken from each sample at the same plane and magnification were analyzed, and the results were averaged. Mitochondrial morphological features, such as location, diversity in morphology, shape, loss of cristae, abnormal matrix density, presence of lipid droplets in the vicinity, autophagic vacuoles and/or electron-dense bodies similar to lysosomes, were evaluated. After outlining the cardiomyocyte border and mitochondria as the cross-sectional area, the mitochondrial density was automatically defined with the iTEM morphometric program (*Olympus*) as a percentage of the analyzed field.

### Oxidative stress and the antioxidant defense system

Tyrosine nitrosylation was used as a marker of the severity of oxidative/nitrosative stress. To estimate extramitochondrial ROS production, NOX-1 and NOX-2 expression was measured. The efficiency of antioxidant defense was estimated by measuring superoxide dismutase, SOD-1, SOD-2 and glutathione peroxidase (GPx) expression.

Hearts were excised immediately after thoracic opening. The free wall of the LV was quickly frozen in liquid nitrogen. LV tissue samples (50 mg) were homogenized in lysis buffer (T-PER Tissue Protein Extraction Reagent, *Thermo Fisher Scientific*) supplemented with protease and phosphatase inhibitor cocktails (*Thermo Fisher Scientific*) using 5 mm stainless steel beads in a TissueLyser LT (*Qiagen*). The homogenate was centrifuged at 14,000×*g* at 4 °C for 10 min, after which the supernatant was collected for further analysis. Total heart protein was measured using the Pierce BCA protein assay kit (*Thermo Fisher Scientific*). Samples containing 20 μg of protein were separated on 4–20% Mini-PROTEAN^®^ TGX™ precast protein gels (*Bio-Rad*) and transferred to nitrocellulose membranes (*Thermo Fisher Scientific*). The membranes were blocked with 5% fat-free milk in Tris-buffered saline containing 0.1% Tween 20. After that, the membranes were incubated overnight with specific primary antibodies against nitrotyrosine (n-tyrosine, 1:2000; *Sigma‒Aldrich*), NADPH oxidase-1 and -2 (NOX-1 and NOX-2; 1:1000; *Sigma‒Aldrich*), superoxide dismutase-1 and -2 (SOD-1 and SOD-2; 1:4000; *Sigma-Aldrich*), glutathione peroxidase (GPx; 1:2000; *Santa Cruz Biotechnology*) and GAPDH (1:2000; *Abcam)*, and secondary antibodies conjugated to horseradish peroxidase (1:2000; *Abcam*). The protein bands were visualized using Westar Supernova enhanced chemiluminescence substrate (*Cyanagen*) and a camera (*UVITEC Ltd. Cambridge*). Densitometric analysis was performed using UVI-Band software (*UVITEC Ltd. Cambridge*). Relative protein levels were normalized to those of GAPDH (Figs. S1, S2).

### Statistical analysis

For normally distributed data, two-way ANOVA followed by the Student Neuman–Keuls test for post-hoc comparisons was used. In the case of a lack of normality, Kruskal‒Wallis one-way ANOVA on ranks followed by Student Neuman–Keuls or Dunn’s post hoc tests were used for all pairwise comparisons. Differences between two groups were detected using the Mann‒Whitney Rank Sum test or Student’s *t* test depending on the normality of the data distribution. Fisher’s exact test was used to compare the occurrence of VT and abnormal Ca^2+^ release among the groups. Differences were considered significant at the level of p < 0.05. The number of hearts in the group was estimated using a sample size test (*SigmaPlot*) assuming 80% test power and a confidence level of p < 0.05.

### Supplementary Information

Supplementary Figure S1.

Supplementary Figure S2.

## Data Availability

All data generated during this study are included in this published article. The datasets used and analyzed during the current study are available from the corresponding author upon reasonable request.

## References

[1] Huikuri HV, Castellanos A, Myerburg RJ (2001). Sudden death due to cardiac arrhythmias. N. Engl. J. Med..

[2] Amuthan R, Curtis AB (2022). Sex-specific considerations in drug and device therapy of cardiac arrhythmias. J. Am. Coll. Cardiol..

[3] Zeitler EP (2022). Arrhythmias in female patients: Incidence, presentation and management. Circ. Res..

[4] Tung R, Zimetbaum P, Josephson ME (2008). A critical appraisal of implantable cardioverter-defibrillator therapy for the prevention of sudden cardiac death. J. Am. Coll. Cardiol..

[5] Germano JJ, Reynolds M, Essebag V, Josephson ME (2006). Frequency and causes of implantable cardioverter-defibrillator therapies: Is device therapy proarrhythmic?. Am. J. Cardiol..

[6] de Vreede-Swagemakers JJM (1997). Out-of-hospital cardiac arrest in the 1990s: A population-based study in the Maastricht area on incidence, characteristics and survival. J. Am. Coll. Cardiol..

[7] Chugh SS (2008). Epidemiology of sudden cardiac death: Clinical and research implications. Prog. Cardiovasc. Dis..

[8] Nattel S, Maguy A, Le Bouter S, Yeh Y-H (2007). Arrhythmogenic ion-channel remodeling in the heart: Heart failure, myocardial infarction, and atrial fibrillation. Physiol. Rev..

[9] Oknińska M, Mączewski M, Mackiewicz U (2022). Ventricular arrhythmias in acute myocardial ischaemia—Focus on the ageing and sex. Ageing Res. Rev..

[10] Bers DM (2006). Altered cardiac myocyte Ca regulation in heart failure. Physiology.

[11] Niggli E (2013). Posttranslational modifications of cardiac ryanodine receptors: Ca^2^^+^ signaling and EC-coupling. Biochim. Biophys. Acta (BBA) Mol. Cell Res..

[12] Danila CI, Hamilton SL (2004). Phosphorylation of ryanodine receptors. Biol. Res..

[13] Lompré AM, Lambert F, Lakatta EG, Schwartz K (1991). Expression of sarcoplasmic reticulum Ca(2+)-ATPase and calsequestrin genes in rat heart during ontogenic development and aging. Circ. Res..

[14] Taffet GE, Tate CA (1993). CaATPase content is lower in cardiac sarcoplasmic reticulum isolated from old rats. Am. J. Physiol. Heart Circ. Physiol..

[15] Campbell SG, Haynes P, Kelsey Snapp W, Nava KE, Campbell KS (2013). Altered ventricular torsion and transmural patterns of myocyte relaxation precede heart failure in aging F344 rats. Am. J. Physiol. Heart Circ. Physiol..

[16] Cooper LL (2013). Redox modification of ryanodine receptors by mitochondria-derived reactive oxygen species contributes to aberrant Ca^2^^+^ handling in ageing rabbit hearts. J. Physiol..

[17] Oknińska M (2021). Effect of age and sex on the incidence of ventricular arrhythmia in a rat model of acute ischemia. Biomed. Pharmacother..

[18] Feridooni HA, Dibb KM, Howlett SE (2015). How cardiomyocyte excitation, calcium release and contraction become altered with age. J. Mol. Cell. Cardiol..

[19] Farrell SR, Ross JL, Howlett SE (2010). Sex differences in mechanisms of cardiac excitation-contraction coupling in rat ventricular myocytes. Am. J. Physiol. Heart Circ. Physiol..

[20] Howlett SE, Grandy SA, Ferrier GR (2006). Calcium spark properties in ventricular myocytes are altered in aged mice. Am. J. Physiol. Heart Circ. Physiol..

[21] Zhang G-Q (2008). Long-term treatment with a Chinese herbal formula, Sheng-Mai-San, improves cardiac contractile function in aged rats: The role of Ca^2^^+^ homeostasis. Rejuvenation Res..

[22] Xu A, Narayanan N (1998). Effects of aging on sarcoplasmic reticulum Ca^2^^+^-cycling proteins and their phosphorylation in rat myocardium. Am. J. Physiol. Heart Circ. Physiol..

[23] Vizgirda VM, Wahler GM, Sondgeroth KL, Ziolo MT, Schwertz DW (2002). Mechanisms of sex differences in rat cardiac myocyte response to β-adrenergic stimulation. Am. J. Physiol. Heart Circ. Physiol..

[24] Terentyev D (2008). Redox modification of ryanodine receptors contributes to sarcoplasmic reticulum Ca^2^^+^ leak in chronic heart failure. Circ. Res..

[25] Belevych AE (2009). Redox modification of ryanodine receptors underlies calcium alternans in a canine model of sudden cardiac death. Cardiovasc. Res..

[26] Adameova A, Shah AK, Dhalla NS (2020). Role of oxidative stress in the genesis of ventricular arrhythmias. Int. J. Mol. Sci..

[27] Miller AA, Drummond GR, Mast AE, Schmidt HHHW, Sobey CG (2007). Effect of gender on NADPH-oxidase activity, expression, and function in the cerebral circulation. Stroke.

[28] Green A, Hossain T, Eckmann DM (2022). Mitochondrial dynamics involves molecular and mechanical events in motility, fusion and fission. Front. Cell Dev. Biol..

[29] Zorov DB, Filburn CR, Klotz L-O, Zweier JL, Sollott SJ (2000). Reactive oxygen species (Ros-induced) Ros release. J. Exp. Med..

[30] Li A (2022). Mitochondrial autophagy: Molecular mechanisms and implications for cardiovascular disease. Cell Death Dis..

[31] Chaudhari SN, Kipreos ET (2018). The energy maintenance theory of aging: Maintaining energy metabolism to allow longevity. BioEssays.

[32] Kander MC, Cui Y, Liu Z (2017). Gender difference in oxidative stress: A new look at the mechanisms for cardiovascular diseases. J. Cell. Mol. Med..

[33] Martínez de Toda I (2023). Sex differences in markers of oxidation and inflammation. Implications for ageing. Mech. Ageing Dev..

[34] Barp J (2002). Myocardial antioxidant and oxidative stress changes due to sex hormones. Braz. J. Med. Biol. Res..

[35] Brandes RP, Mügge A (1997). Gender differences in the generation of superoxide anions in the rat aorta. Life Sci..

[36] Chen Y (2012). Evaluation of gender-related differences in various oxidative stress enzymes in mice. Chin. J. Physiol..

[37] Koebele SV, Bimonte-Nelson HA (2016). Modeling menopause: The utility of rodents in translational behavioral endocrinology research. Maturitas.

[38] Scherer NM, Deamer DW (1986). Oxidation of thiols in the Ca^2^^+^-ATPase of sarcoplasmic reticulum microsomes. Biochim. Biophys. Acta (BBA) Biomembr..

[39] Tellez JO (2011). Ageing-dependent remodelling of ion channel and Ca^2^^+^ clock genes underlying sino-atrial node pacemaking. Exp. Physiol..

[40] Morita N (2009). Increased susceptibility of aged hearts to ventricular fibrillation during oxidative stress. Am. J. Physiol. Heart Circ. Physiol..

[41] Rohr S, Kucera JP, Fast VG, Kléber AG (1997). Paradoxical improvement of impulse conduction in cardiac tissue by partial cellular uncoupling. Science.

[42] Curtis MJ (2013). The Lambeth Conventions (II): Guidelines for the study of animal and human ventricular and supraventricular arrhythmias. Pharmacol. Ther..

[43] Mackiewicz U, Emanuel K, Lewartowski B (2000). Dihydropyridine receptors functioning as voltage sensors in cardiac myocytes. J. Physiol. Pharmacol..

[44] Choi HS, Eisner DA (1999). The role of sarcolemmal Ca^2^^+^-ATPase in the regulation of resting calcium concentration in rat ventricular myocytes. J. Physiol..

