# Supplementary figures and images for: Sex- and age-dependent susceptibility to ventricular arrhythmias in the rat heart ex vivo
Source: Sci Rep. 2024 Feb 11;14:3460. doi: 10.1038/s41598-024-53803-9 (PMC10859380; doi:10.1038/s41598-024-53803-9)

a

**NOX-1**

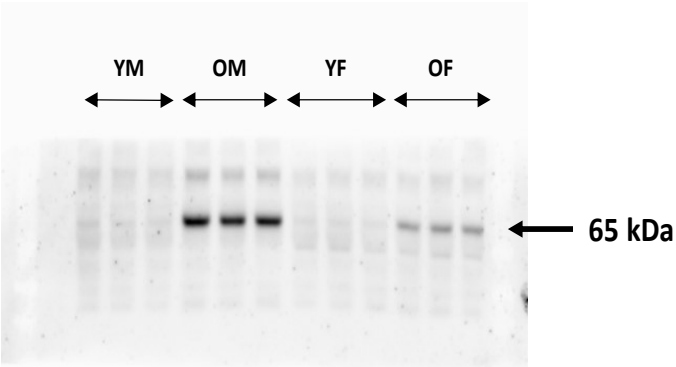

**GAPDH**

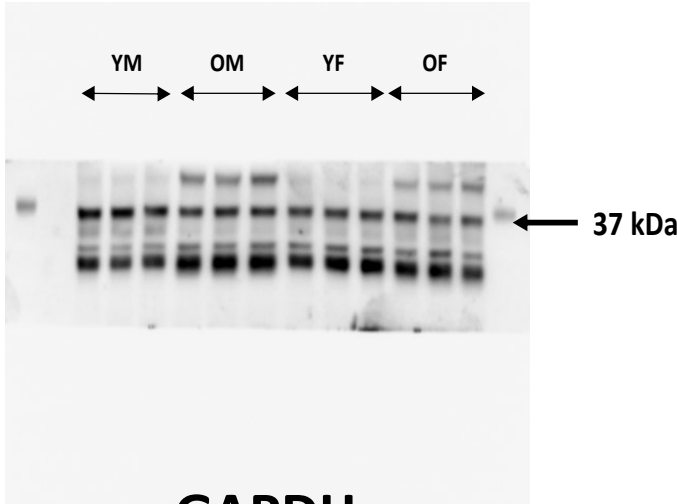

b

**NOX-2**

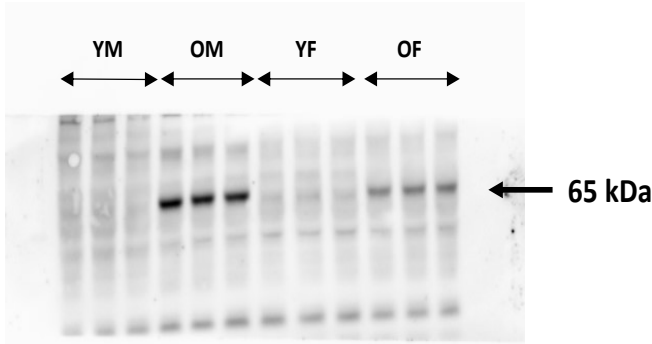

**GAPDH**

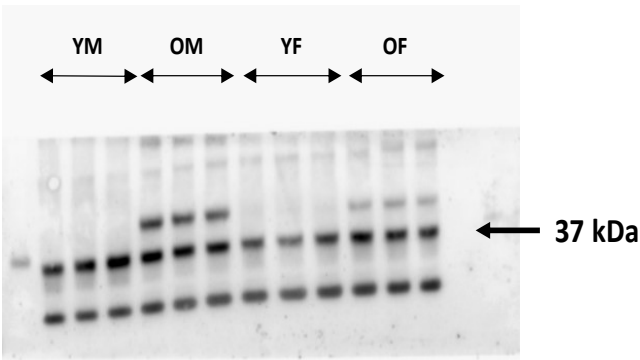

c

**n-tyrosine**

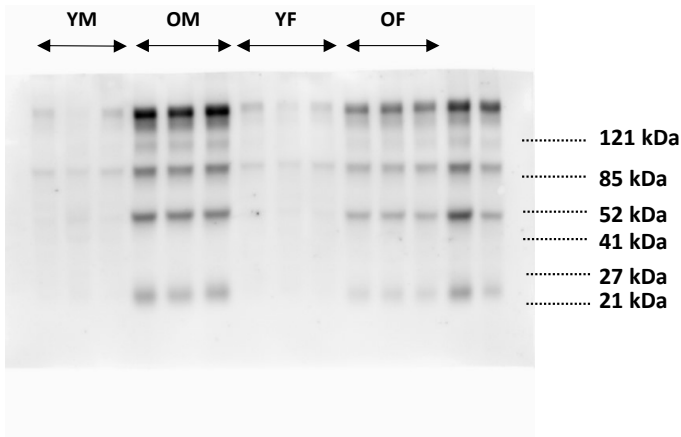

**GAPDH**

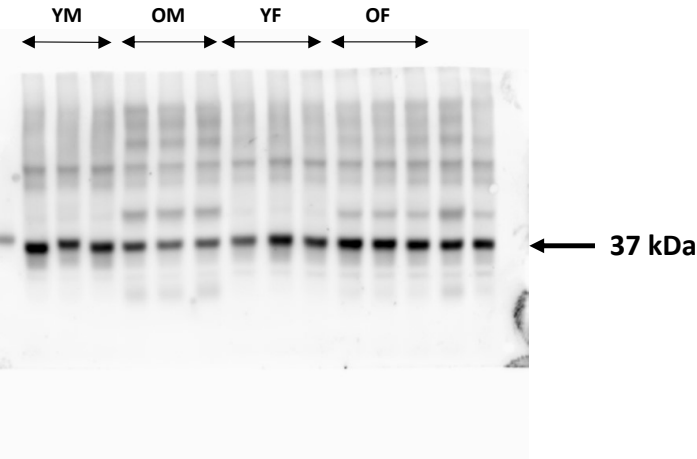

Supplement: Supplementary file 1 — Supplementary Figure S1. [file 41598_2024_53803_MOESM1_ESM.pdf]

a

SOD-1

GAPDH

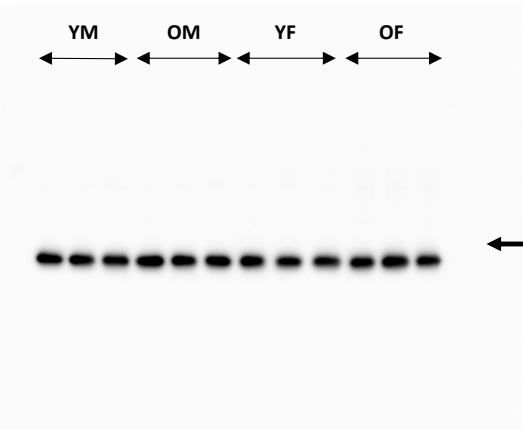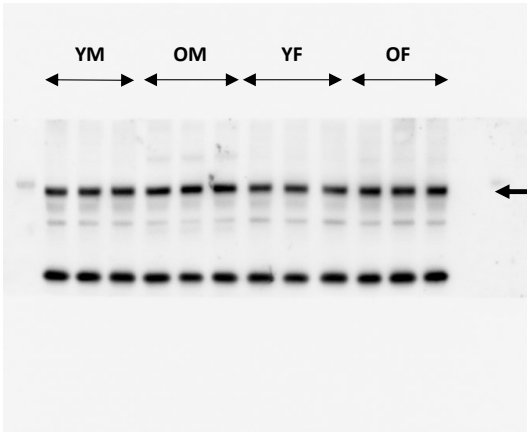

b

SOD-2

GAPDH

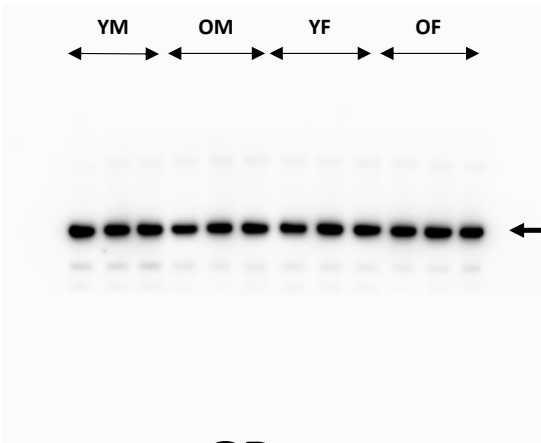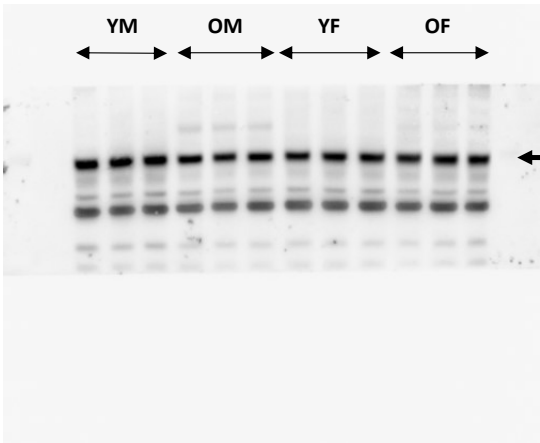

c

GPx

GAPDH

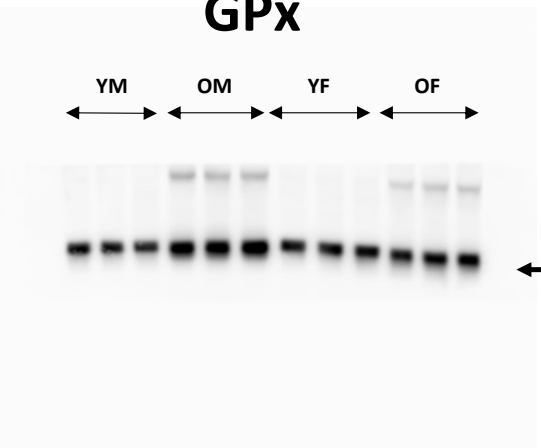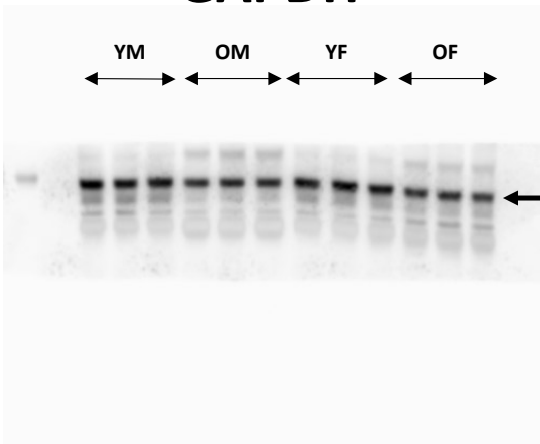

Supplement: Supplementary file 2 — Supplementary Figure S2. [file 41598_2024_53803_MOESM2_ESM.pdf]
